# Fano and Electromagnetically Induced Transparency Resonances in Dual Side-Coupled Photonic Crystal Nanobeam Cavities

**DOI:** 10.3390/ma17246213

**Published:** 2024-12-19

**Authors:** Yong Zhao, Yuxuan Chen, Lijun Hao

**Affiliations:** 1 School of Science, Jiangsu Provincial Research Center of Light Industrial Optoelectronic Engineering and Technology, Jiangnan University, Wuxi 214122, China; 8202202011@jiangnan.edu.cn; 2 Department of Electronic Engineering, School of Internet of Things Engineering, Jiangnan University, Wuxi 214122, China; 3 Wuxi Suntech Power Company Limited, Wuxi 214208, China; 4 Key Laboratory of Intelligent Optical Sensing and Manipulation of the Ministry of Education, National Laboratory of Solid State Microstructures, College of Engineering and Applied Sciences, Institute of Optical Communication Engineering, Nanjing University, Nanjing 210093, China; 652023340002@smail.nju.edu.cn; 5 College of Mathematics and Physics, Nanjing Tech University, Nanjing 211816, China

**Keywords:** Fano resonance, EIT resonance, nanobeam cavity

## Abstract

We propose two types of structures to achieve the control of Fano and electromagnetically induced transparency (EIT) line shapes, in which dual one-dimensional (1D) photonic crystal nanobeam cavities (PCNCs) are side-coupled to a bus waveguide with different gaps. For the proposed type Ⅰ and type Ⅱ systems, the phase differences between the nanobeam periodic structures of the two cavities are *π* and 0, respectively. The whole structures are theoretically analyzed via the coupled mode theory and numerically demonstrated using the three-dimensional finite-difference time-domain (3D FDTD) method. The simulation results show that the proposed structure can achieve several kinds of spectra, including Fano, EIT and asymmetric EIT line shapes, which is dependent on the width of the bus waveguide. Compared to the previously proposed Fano resonator with 1D PCNCs, the proposed structures have the advantages of high transmission at the resonant peak, low insertion loss at non-resonant wavelengths, a wide free spectral range (FSR) and a high roll-off rate. Therefore, we believe the proposed structure can find broad applications in optical switches, modulators and sensors.

## 1. Introduction

Fano resonance, which is induced by the interference between a discrete mode and a continuum mode, has attracted considerable interest from researchers [1,2,3,4,5,6,7,8]. Compared to the usual Lorentzian resonance with a symmetric line shape, Fano resonance can build a much steeper asymmetric line shape. By adjusting the frequency difference between the discrete mode and the continuum mode to zero, Fano resonance can be converted to electromagnetically induced transparency (EIT) resonance with a steep symmetric line shape. Due to the steep line shape, Fano and EIT resonances can find broad applications in the field of photonic integration, including optical filtering, switching, modulating and sensing [4,9,10,11,12,13].

Micro-ring, micro-disk and photonic crystal cavities are the most commonly used resonators for achieving on-chip Fano resonance. Among them, one-dimensional (1D) photonic crystal nanobeam cavities (PCNCs) have the advantages of compact size, small mode volume, easy integration and easy fabrication and thus have attracted extensive attention recently [14,15,16,17,18,19,20,21]. One of the commonly used regimes for achieving Fano resonance is making the 1D PCNC side-coupled to the bus waveguide with one or several holes [22,23,24,25,26,27,28,29]. In this regime, the 1D PCNC is used to excite a high Q mode, i.e., a discrete mode, while the holes in the bus waveguide can excite a low Q mode, i.e., a continuum mode. The major drawback of this is that the holes in the bus waveguide will bring apparent transmission loss at off-Fano resonance wavelengths or even lead to unwanted strong resonances at other wavelengths. Therefore, its application is restricted. For example, it cannot be used in a WDM system as a wavelength-selective optical switch. Except for etching holes on the bus waveguide, several dual coupled PCNC systems have been proposed. F. Jiang et al. proposed one EIT resonance structure in which one PCNC is simultaneously coupled to the bus waveguide and another identical PCNC [30]. However, it strictly belongs to Autler–Townes splitting (ATS) resonance, since the Q-factors of the two PCNCs are nearly the same. And thus, its line width is very wide. J. Ma proposed an indirectly coupled PCNC–waveguide system, but its line width is also wide in their design [31].

Instead of etching holes on the bus waveguide, we proposed a design in which the bus waveguide is side-coupled to two 1D PCNCs with different waveguide gaps in this paper. Two resonances with different Q-factors can be formed because of the different coupling coefficients between the PCNCs and the bus waveguide. Here, two types of dual-PCNC systems are studied. Except for the temporary coupled mode theory (CMT), steady-state coupled mode equations with differential forms of the electric field along the propagation direction are also used to model the entire system to investigate the impacts of structural parameters. These equations predict that the line shape of the transmission spectrum can be significantly influenced by the width of the waveguide. Then, the three-dimensional finite-difference time-domain (3D FDTD) method is used to simulate the performances of the systems. This method proves that by adjusting the position of the air holes and the width of the bus waveguide, the proposed structure can achieve several kinds of spectra including symmetric EIT, Fano and asymmetric EIT line shapes. Compared with the PCNC system with holes in the bus waveguide, the proposed structure has the advantages of a wide free spectral range (FSR) and transparency at non-resonant wavelengths. The proposed structure with an EIT line shape has a high Q-factor and can be further used as an optical sensor or filter, while the proposed structures with Fano and asymmetric EIT line shapes can achieve high extinction ratios and high roll-off rates and can be further used as optical switches or modulators.

## 2. Theoretical Model and Analysis

**Figure 1:**
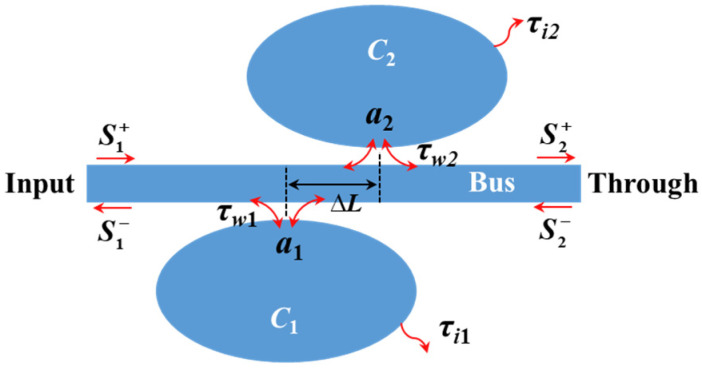
Simplified model of two side-coupled standing-wave cavities (*C*_1_ and *C*_2_).

A PCNC is a standing-wave cavity in which the resonant mode decays equally into the forward and backward propagating modes in the bus waveguide [32]. A simplified model of two side-coupled standing-wave cavities (*C*_1_ and *C*_2_) is schematically illustrated in Figure 1. Here, *a* represents the electric fields of the cavities, and 1/*τ_i_* and 1/*τ_w_* are the decay rates caused by the intrinsic loss and waveguide coupling loss, respectively. The subscripts ‘1’ and ‘2’ of *a*, *τ_i_* and *τ_w_* refer to *C*_1_ and *C*_2_, respectively. The Q-factors related to *τ_i_* and *τ_w_* are expressed as *Q_i_* = *ω*_0_*τ_i_*/2 and *Q_w_* = *ω*_0_*τ_w_*/2, respectively, where *ω*_0_ is the resonant frequency. $S_{l}^{+}$ and $S_{l}^{-}$ are the forward and backward waves at the left port, respectively. $S_{r}^{+}$ and $S_{r}^{-}$ are the forward and backward waves at the right port, respectively. In this case, the two standing-wave cavities are indirectly coupled to each other through the bus waveguide. According to the temporary CMT, when the light is launched only from the left port (i.e., $S_{r}^{-}$ = 0), the evaluations of *a*_1_ and *a*_2_ can be expressed as follows [33]:

(1) $$\frac{da_{1}}{dt}=(-i\omega_{1}-\frac{1}{\tau_{i1}}-\frac{1}{\tau_{w1}})a_{1}+\sqrt{\frac{1}{\tau_{w1}}}S_{l}^{+}-\sqrt{\frac{1}{\tau_{w1}\tau_{w2}}}e^{-i\Delta \phi }a_{2}$$

(2) $$\frac{da_{2}}{dt}=(-i\omega_{2}-\frac{1}{\tau_{i2}}-\frac{1}{\tau_{w2}})a_{1}+\sqrt{\frac{1}{\tau_{w1}}}e^{-i\Delta \phi }S_{l}^{+}-\sqrt{\frac{1}{\tau_{w1}\tau_{w2}}}e^{-i\Delta \phi }a_{1}$$

Here, *ω*_1_ (=2*πc*/*λ*_1_) and *ω*_2_ (=2*πc*/*λ*_2_) are the resonant frequencies of *C*_1_ and *C*_2_, respectively, where *λ*_1_ and *λ*_2_ are the resonant wavelengths. ∆*ϕ* is the phase shift between the waveguide couplings of *C*_1_ and *C*_2_, which can be expressed as follows:

(3) $$\Delta \phi =\phi_{2}-\phi_{1}+\beta_{\mathrm{bus}}\Delta L$$

where *ϕ*_1_ and *ϕ*_2_ are the waveguide coupling phases of *C*_1_ and *C*_2_, respectively; *β*_bus_ is the wavevector in the bus waveguide; and ∆*L* is the distance between *C*_1_ and *C*_2_. According to the law of energy conservation, when a time-harmonic wave with a frequency of *ω* inputs from the left port, the normalized amplitudes of reflection and transmission can be respectively expressed as follows:

(4) $$S_{l}^{-}=-\sqrt{\frac{1}{\tau_{w1}}}a_{1}-\sqrt{\frac{1}{\tau_{w2}}}a_{2}e^{i\Delta \phi }$$

(5) $$S_{r}^{+}=1-\sqrt{\frac{1}{\tau_{w1}}}a_{1}-\sqrt{\frac{1}{\tau_{w2}}}a_{2}e^{i\Delta \phi }$$

In the steady state, when light with a frequency of *w* is launched into the input port, it can be obtained that *da*_1_/*dt* = *iωa*_1_ and *da*_2_/*dt* = *iωa*_1_. Thus, Equations (1) and (2) can be solved, and then the transmission and reflection amplitudes can be obtained.

**Figure 2:**
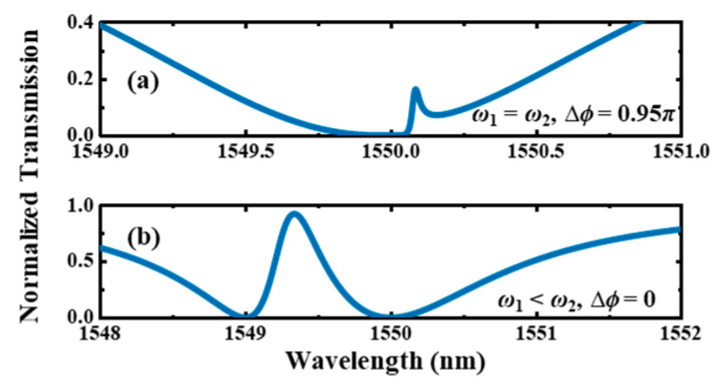
The calculated transmission spectra of the (**a**) type Ⅰ and (**b**) type Ⅱ systems based on the temporary CMT. The coupling Q-factors are *Q_w_*_1_ = 500 and *Q_w_*_2_ = 2000, and the intrinsic Q-factors are *Q_i_*_1_ = *Q_i_*_2_ = 1 × 10^5^. The resonant wavelengths of *C*_1_ and *C*_2_ are *λ*_1_ = *λ*_2_ = 1550 nm in (**a**) and *λ*_1_ = 1550 nm and *λ*_2_ = 1549 nm in (**b**).

When only one traveling wave resonator is sided-coupled to the bus, light at the resonant wavelength can be reflected to the input port, resulting in a dip in the transmission spectrum at the through port. By varying ∆*ϕ*, complex interference phenomena can be generated, leading to a non-Lorentzian line shape. Two systems are analyzed in this paper. In the type Ⅰ system, *C*_1_ and *C*_2_ are identical, i.e., *ω*_1_ = *ω*_2_, and *τ_i_*_1_ = *τ_i_*_2_. And there is *τ_w_*_1_ > *τ_w_*_2_ to form two resonances with different Q-factors. ∆*ϕ* is set close to *π* so that the reflected lights near the resonant wavelength caused by *C*_1_ and *C*_2_ can interfere destructively in the bus waveguide. In the type Ⅱ system, the resonances with two different Q-factors have a slightly resonant frequency difference; there is *ω*_1_ > *ω*_2_, *τ_i_*_1_ = *τ_i_*_2_, *τ_w_*_1_ > *τ_w_*_2_ and ∆*ϕ* = 0. Figure 2a,b show the calculated transmission spectra of the type Ⅰ and type Ⅱ systems, respectively, with the coupling Q-factors of *Q_w_*_1_ = 500 and *Q_w_*_2_ = 2000 and intrinsic Q-factors of *Q_i_*_1_ = *Q_i_*_2_ = 1 × 10^5^. Here, the dispersion of ∆*ϕ* is neglected for simplicity. The resonant wavelengths of *C*_1_ and *C*_2_ of the type Ⅰ system are both 1550 nm. The resonant wavelengths of *C*_1_ and *C*_2_ of the type Ⅱ system are 1550 nm and 1549 nm, respectively. Both systems can achieve a high Q mode near 1550 nm. For the type Ⅰ system, an infinite Q-factor can be theoretically achieved when ∆*ϕ* equals *π*.

The temporary CMT mainly focuses on the energy transfer of the resonant system without considering its geometric shapes and coupling ways. In order to obtain the influence of the structural parameters, we employ another type of coupled mode equation which involves the differential form of the electric field along the propagation direction in the steady state, which is commonly used in the study of 1D periodic structures such as PCNCs and Bragg gratings. The coupled mode equations can be expressed as follows [34,35]:

(6) $$\frac{dF_{0}(z)}{dz}=\;(\alpha_{0}-i\delta_{0})F_{0}+i\mu_{01}F_{1}+i\mu_{02}F_{2},\;-\frac{dR_{0}(z)}{dz}=\;(\alpha_{0}-i\delta_{0})R_{0}+i\mu_{01}R_{1}+i\mu_{02}R_{2}$$

(7) $$\frac{dF_{1}(z)}{dz}=\;(\alpha_{1}-i\delta_{1})F_{1}+i\mu_{01}F_{0}+i\kappa_{11}R_{1},\;-\frac{dR_{1}(z)}{dz}=\;(\alpha_{1}-i\delta_{1})R_{1}+i\mu_{01}R_{0}+i\kappa_{11}F_{1}$$

(8) $$\frac{dF_{2}(z)}{dz}=\;(\alpha_{2}-i\delta_{2})F_{2}+i\mu_{02}F_{2}+i\kappa_{22}R_{2},\;-\frac{dR_{2}(z)}{dz}=\;(\alpha_{2}-i\delta_{2})R_{2}+i\mu_{02}R_{0}+i\kappa_{22}F_{2}$$

where the subscripts ‘0’, ‘1’ and ‘2’ refer to the fundamental transverse electric (TE_0_) modes of the bus waveguide, *C*_1_ and *C*_2_, respectively. *F* and *R* represent the amplitudes of the forward and backward propagating waves, respectively; *α* is the waveguide loss; *δ* is the detuning factor; *μ* is the directional coupling coefficient between different transverse modes; and *κ* is the contra-directional coupling coefficient between the forward and backward transverse modes in the cavity. The detuning factor can be expressed as follows:

(9) $$\delta_{i}=n_{eff\;i}\frac{2\pi }{\lambda }-\frac{\pi }{\Lambda }\;(i=0,\;1,\;2)$$

where *n_eff i_* is the effective index of the transverse mode, and *λ* is the wavelength. For non-uniform structures such as the phase-shifted grating, the coupled mode equations can be solved using the transfer matrix method. The transfer matrix of a subsection of length ∆*z* can be expressed as follows [35,36]:

(10) $$E(z+\Delta z)=e^{A\Delta z}E(z)$$

where *E* is given by

(11) $$E(z)={\left[\begin{array}{cccccc} F_{0}(z) & R_{0}(z) & F_{1}(z) & R_{1}(z) & F_{2}(z) & R_{2}(z) \end{array}\right]}^{T}$$

and *A* is a 6 × 6 matrix which is given by

(12) $$A=\left[\begin{array}{cccccc} \alpha_{0}-i\delta_{0} & 0 & -i\mu_{01} & 0 & -i\mu_{02} & 0\\ 0 & -(\alpha_{0}-i\delta_{0}) & 0 & i\mu_{01} & 0 & i\mu_{02}\\ -i\mu_{01} & 0 & \alpha_{1}-i\delta_{1} & i\kappa_{11} & 0 & 0\\ 0 & i\mu_{01} & -i\kappa_{11} & -(\alpha_{1}-i\delta_{1}) & 0 & 0\\ -i\mu_{02} & 0 & 0 & 0 & \alpha_{2}-i\delta_{2} & i\kappa_{22}\\ 0 & i\mu_{02} & 0 & 0 & -i\kappa_{22} & -(\alpha_{2}-i\delta_{2}) \end{array}\right]$$

The transfer matrix of the entire structure (*T*) can be obtained by dividing the structure into several subsections in the *z* direction and integrating the functions of all the subsections. When there are *m* subsections, *T* can be expressed as follows:

(13) $$T=e^{A_{m}\Delta z_{m}}\cdot \cdot \cdot e^{A_{2}\Delta z_{2}}\cdot e^{A_{1}\Delta z_{1}}$$

where the subscripts ‘1’, ‘2’ and ‘*m*’ refer to the first, second and *m*th subsections, respectively, and the subsections are numbered sequentially from left to right (i.e., from the input port to the through port). The relationship between the amplitudes of the waves can be expressed as E(L)=T⋅E(0), where *L* is the length of the entire structure. The boundary conditions are given by *F*_0_(0) = 1, *F*_1_(0) = *R*_1_(0) = *F*_2_(0) = *R*_2_(0) = 0 at the input port and *R*_0_(*L*) = *F*_1_(*L*) = *R*_1_(*L*) = *F*_2_(*L*) = *R*_2_(*L*) = 0 at the through port. Then, the normalized transmission of the entire structure at the through port can be obtained.

**Figure 3:**
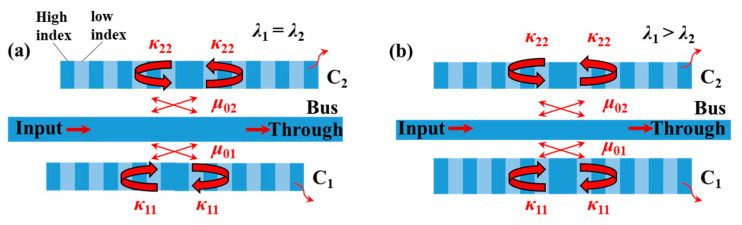
Models of two side-coupled cavities with phase-shifted periodic structure under weak perturbation conditions: (**a**) Type Ⅰ system with same resonant wavelengths and grating phase difference of *π* between *C*_1_ and *C*_2_. (**b**) Type Ⅱ system with different resonant wavelengths and same grating phase between *C*_1_ and *C*_2_.

Next, we consider an ideal case in which the two resonators provide weak refractive index modulations (i.e., the perturbation is weak), and a *π* phase shift is inserted in the middle of each resonator, as shown in Figure 3. In this situation, the resonance of each cavity is strictly at the Bragg wavelength. In the following calculations, the effective index of *C*_1_ is 2.2 (*n_eff_*_1_ = 2.2), and the perturbation period Λ is set to 352 nm to achieve a resonant wavelength of *C*_1_ at 1550 nm. The waveguide dispersion is neglected. The modal losses are *α*_0_ = −11.5 m^−1^ and *α*_1_ = *α*_2_ = −1.15 m^−1^, which correspond to a −1 dB/cm waveguide loss in the bus waveguide and a −10 dB/cm waveguide loss in both *C*_1_ and *C*_2_.

**Figure 4:**
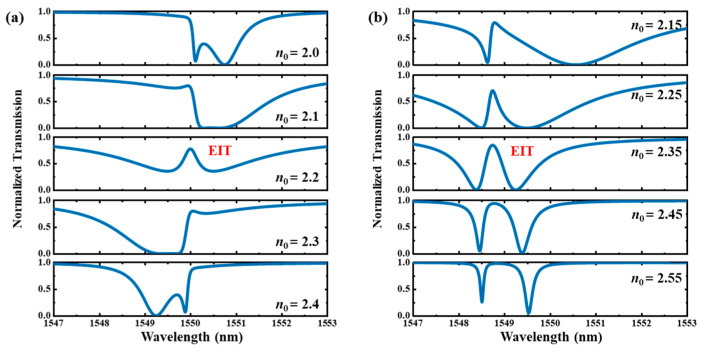
The calculated transmission spectra of the (**a**) type Ⅰ and (**b**) type Ⅱ systems under weak perturbation conditions based on the coupled mode equations of Equations (6)–(8).

For the type Ⅰ system, the coupling coefficients are *μ*_01_ = 2 × 10^4^ m^−1^, *μ*_02_ = 6 × 10^4^ m^−1^ and *κ*_11_ = *κ*_22_ = 8 × 10^5^ m^−1^. The cavity length and grating period of *C*_1_ and *C*_2_ are both 15 μm and 352 nm, respectively. The effective indices of *C*_1_ and *C*_2_ are set as *n_eff_*_1_ = *n_eff_*_2_ = 2.2, and the deviation between *C*_1_ and *C*_2_ (∆*L*) is Λ/2 so that ∆*ϕ* equals *π*. The calculated spectra of the type Ⅰ system with different effective indices of the bus waveguide are shown in Figure 4a. This figure shows that an EIT line shape is achieved when *n_eff_*_0_ = *n_eff_*_1_ = *n_eff_*_2_ = 2.2. When the value of *n_eff_*_0_ deviates from 2.2, the Fano resonance line shape with a sharp edge emerges at 1550 nm. Specifically, the slope of the sharp edge is negative when *n_eff_*_0_ is less than 2.2 and is positive when n*_eff_*_0_ is larger than 2.2.

For the type Ⅱ system, the grating phases of *C*_1_ and *C*_2_ are identical (∆*ϕ* = 0). The effective indices of *C*_1_ and *C*_2_ are *n_eff_*_1_ = 2.2 and *n_eff_*_2_ = 2.198, respectively, to achieve slightly different resonant wavelengths. The other parameters are all the same as those of the type Ⅰ system. The calculated spectra of the type Ⅱ system with different effective indices of the bus waveguide are shown in Figure 4b. This figure shows that an EIT line shape is achieved when *n_eff_*_2_ = 2.35. Compared with the type Ⅰ system, its dip can reach a minimum value near 0. When the value of *n_eff_*_0_ deviates from 2.35, the so-called asymmetric–EIT or Fano–EIT resonance line shape emerges. The transmission peak with a flat top and a wide bandwidth occurs when *n_eff_*_0_ is larger than 2.25.

## 3. Design and Simulation

**Figure 5:**
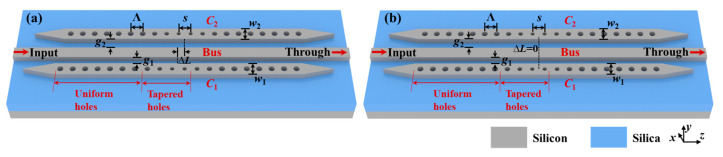
Schematics of proposed (**a**) type Ⅰ and (**b**) type Ⅱ dual-PCNC systems on SOI platform.

**Figure 6:**
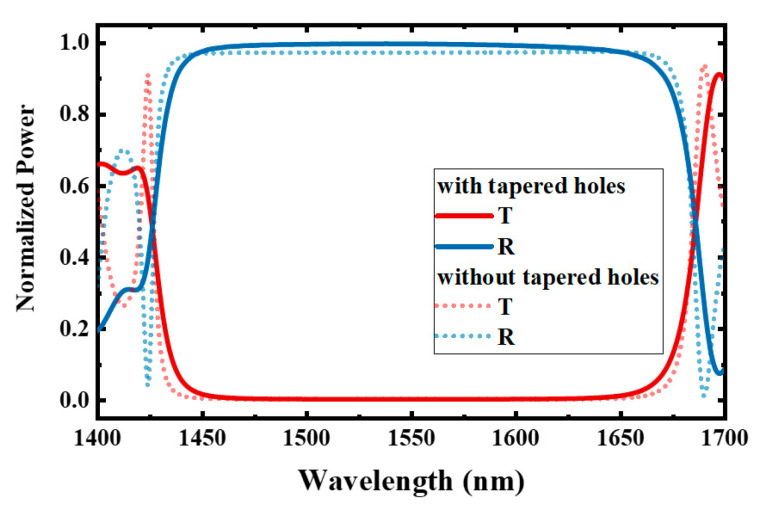
The reflection spectra of the designed Bragg reflector with 4 tapered holes and 9 uniform holes and the Bragg reflector with 13 uniform holes.

To verify the results of the above-analyzed coupled resonators based on periodic structures, we designed two types of dual-PCNC systems on an SOI platform, as shown in Figure 5. The thickness of the top silicon layer (*n* = 3.476) is 220 nm, and the upper and lower claddings are both silica (*n* = 1.444). Two 1D PCNCs (*C*_1_ and *C*_2_) are side-coupled to one strip bus waveguide with gaps denoted as *g*_1_ and *g*_2_. The widths of the bus waveguide, *C*_1_ and *C*_2_ are denoted as *w*_0_, *w*_1_ and *w*_2_, respectively. The PCNC contains two identical Bragg reflectors. The center-to-center distance between the neighboring holes, i.e., the period, is denoted as Λ. The hole radius is denoted as *r*. Each Bragg reflector consists of 13 holes. The center-to-center distance between the first holes of the reflectors on each side (denoted as *s*) is 495 nm. The radii of the first four holes (*r*_1_~*r*_4_) are linearly tapered from 50 nm to 80 nm to reduce scattering loss, and the radius of the other nine holes is 90 nm. The Λ of the uniform holes is 392 nm, while the Λ of the first four holes are 355 nm, 364 nm, 374 nm and 383 nm, respectively, to maintain the Bragg wavelength constant at 1550 nm for all holes. Figure 6 shows the reflection spectra of the designed Bragg reflector with tapered holes and the Bragg reflector with 13 uniform holes using the 3D FDTD method. In the FDTD simulation, the grid size for meshing is ∆*x* = ∆*y* = ∆*z* = 24 nm, and the perfectly matched layer (PML) conditions are applied at the boundaries to absorb the transmitted and scattered light. In addition, the spacing between the waveguide core and the boundary is 490 nm in the *y* direction and 675 nm in the *x* direction.

**Figure 7:**
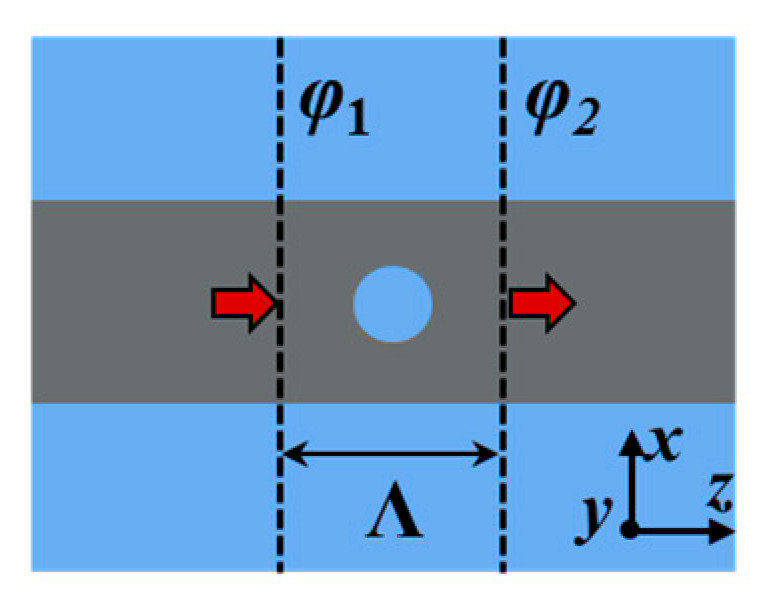
A schematic of the calculation of the effective index of the strip waveguide with a hole.

This shows that the tapered holes can efficiently decrease the insertion loss, and a high reflection of 0.996 is achieved at 1550 nm. The calculated group index in the 450 nm strip waveguide is 4.332. When the holes with radii of 50 nm, 60 nm, 70 nm, 80 nm and 90 nm are etched, the group indices (*n_g_*) at 1550 nm are 3.853, 3.730, 3.587, 3.441 and 3.291, respectively. These values are obtained by simulating the phase change of light traveling through a photonic crystal period via the FDTD method, as shown in Figure 7 [35]. Here, *φ*_1_ and *φ*_2_ are the phases of light at the left and right boundaries, respectively. The effective index (*n_eff_*) at *λ* satisfies

(14) $$n_{eff}(\lambda )\frac{2\pi }{\lambda }\Lambda =\varphi_{2}(\lambda )-\varphi_{1}(\lambda )$$

Then, the group index can be calculated using *n_g_* (*λ*) = *n_eff_* (*λ*) − *λ dn_eff_/dλ*.

**Figure 8:**
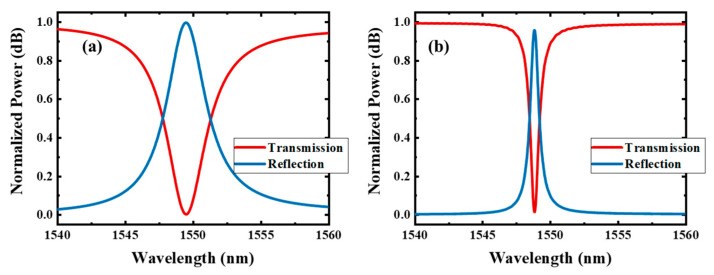
Simulated (**a**) transmission and (**b**) reflection spectra when only *C*_1_ or *C*_2_ is side-coupled to the bus waveguide.

For the type Ⅰ dual-PCNC system, *w*_0_, *w*_1_ and *w*_2_ are designed to be 406.8 nm, 450 nm and 450 nm, respectively; *s*, *g*_1_ and *g*_2_ are 188 nm, 150 nm and 250 nm, respectively. The simulated transmission and reflection spectra when only *C*_1_ or *C*_2_ is side-coupled to the bus waveguide are shown in Figure 8a,b, respectively. Due to the difference in waveguide gaps, there is a slight discrepancy in the resonant wavelengths of *C*_1_ and *C*_2_, which are 1549.5 nm and 1548.9 nm, respectively. The loaded Q-factors of *C*_1_ and *C*_2_ are 433 and 2067, respectively. The intrinsic Q-factor of the cavity is deduced to be 1.0 × 10^5^.

**Figure 9:**
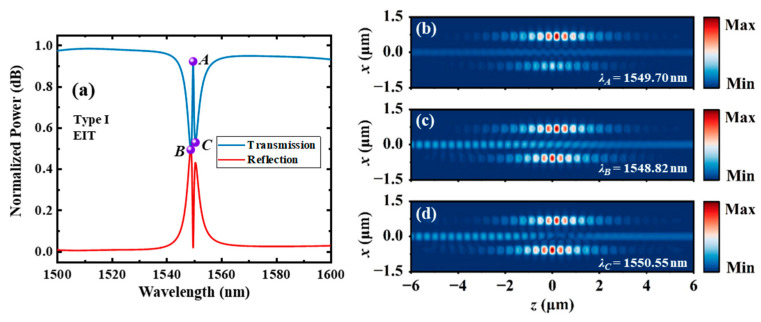
(**a**) The transmission and reflection spectra of the type Ⅰ dual-PCNC system with an EIT-like line shape, and the |*H_y_*| profile at the (**b**) transmission peak point *A*, (**c**) transmission dip point *B* and (**d**) transmission dip point *C* calculated by the 3D FDTD method.

The calculated transmission and reflection spectra of the entire system using the 3D FDTD method are shown in Figure 9a. The simulation time is set to a large value of 30 ps to allow the resonance to reach the steady state. A narrow symmetric transparency window can be observed near the resonant wavelengths of *C*_1_ and *C*_2_ by eliminating the resonant absorption. The transmission, extinction ratio, 3 dB bandwidth and Q-factor of the EIT-like peak are 0.92, 2.35 dB, 0.57 nm and 2719, respectively. In addition, its FSR is wider than 50 nm. The points of the transmission peak and two dips are labeled as *A*, *B* and *C*, respectively. Figure 9b–d show the distributions of magnetic fields (|*H_y_*|) at points *A*, *B* and *C*, respectively. This figure shows that the energy of light is stored almost evenly in the two resonant cavities at points *B* and *C*. Meanwhile, the energy of light is mainly stored in the high Q cavity (*C*_2_) at point *A*.

As previously stated, the resonant line shape can be changed by varying the effective index of the bus waveguide to further control ∆*ϕ*. This purpose can be achieved by varying the width of the bus waveguide. Figure 9a shows the transmission spectra of the type Ⅰ system with different *w*_0_. This figure shows that the transmission line shape is greatly dependent on *w*_0_. According to the Fano theory, the line shape can be described by the Fano formula *T* = 1 − (*q* + Ω)^2^/(1 + Ω^2^), where Ω is the reduced frequency, and *q* is the asymmetric parameter [37]. When *w*_0_ increases to 435 nm, it exhibits an apparently Fano line shape near 1549.2 nm, with a fitted *q* of 1.86. And a sharp edge occurs with a high roll-off rate of about −96.9 dB/nm and a high extinction ratio of 14.4 dB. However, when *w*_0_ decreases to 375 nm, the line shape only becomes somewhat asymmetric, and the Fano line shape with a sharp edge does not occur. The fitted *q* is only 0.49. In addition, the coupling between the cavity and bus waveguide becomes larger as *w*_0_ decreases, which makes the line width much wider. It should be noted that the trend in the line shape caused by the effective index variation in the bus waveguide is opposite to the one described in Section 2. This disparity may be due to the non-uniform refractive index distributions of the two cavities with tapered holes. Thus, the coupling phase between the cavity and bus waveguide is complicated, which will significantly influence ∆*ϕ*.

**Figure 10:**
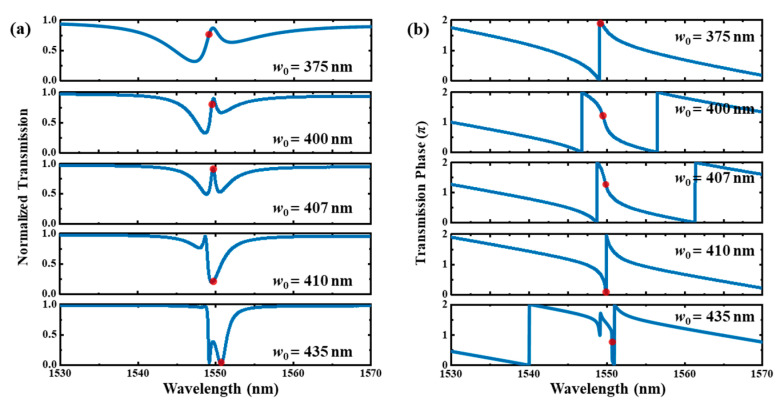
(**a**) Transmission spectra and (**b**) phase of type Ⅰ dual-PCNC system with different bus waveguide widths calculated by 3D FDTD method. Red dot is point of maximum slope change in phase relative to wavelength.

The calculated transmission phases with different *w*_0_ are shown in Figure 10b. The red dot indicates the point of the maximum slope change in phase relative to wavelength, which implies a positive group delay corresponding to the slow light effect. When the spectrum has a perfectly symmetric EIT-like line shape, the highest group delay point is located at the transmission peak, and the calculated group delay is 8.3 ps. When the line shape becomes asymmetric, this point deviates from the transmission peak.

**Figure 11:**
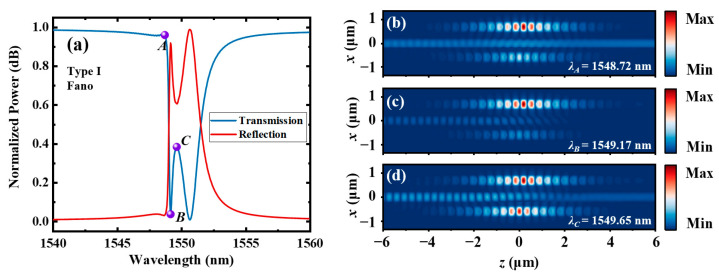
(**a**) The transmission and reflection spectra of the type Ⅰ dual-PCNC system with a Fano line shape (*w*_0_ = 435 nm), and the |*H_y_*| profile at (**b**) point *A*, (**c**) point *B* and (**d**) point *C* calculated by the 3D FDTD method.

The |*H_y_*| distributions of the type Ⅰ dual-PCNC system with a Fano line shape (*w*_0_ = 435 nm) are also calculated. Figure 11b, c and d, respectively, show the |*H_y_*| distributions at points *A*, *B* and *C*, which are labeled in Figure 11a. This figure shows that the energy of light is mainly stored in *C*_2_ at points *A* and *B* and is almost evenly stored in the two resonant cavities at point *C*.

**Figure 12:**
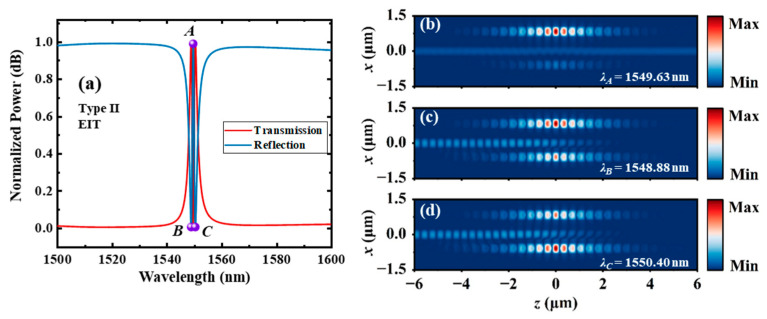
(**a**) The transmission and reflection spectra of the type Ⅱ dual-PCNC system with an EIT-like line shape, and the |*H_y_*| profile at the (**b**) transmission peak point *A*, (**c**) transmission dip point *B* and (**d**) transmission dip point *C* calculated by the 3D FDTD method.

For the type Ⅱ dual-PCNC system, ∆*L* is 0, and *g*_1_ and *g*_2_ are designed to be 100 μm and 400 μm, respectively. The other parameters are the same as those of the type Ⅰ system. A resonant wavelength difference of 0.6 nm between *C*_1_ and *C*_2_ are achieved by using the different waveguide gaps. The loaded Q-factors of *C*_1_ and *C*_2_ are 455 and 15,000, respectively. The calculated transmission and reflection spectra of the entire system using the 3D FDTD method are shown in Figure 12a. A narrow EIT-like transmission peak with a 3 dB bandwidth of 0.69 nm occurs at 1549.6 nm, corresponding to a Q-factor of 2246. A high transmission of 0.989 and a high extinction ratio of 25.2 dB are also achieved. The points of the transmission peak and two dips are labeled as *A*, *B* and *C*, respectively. Figure 11b–d show the |*H_y_*| distributions at points *A*, *B* and *C*, respectively. It can be seen that the energy of light is more concentrated in *C*_2_ at point *B* and is more concentrated in *C*_1_ at point *C*, since the resonant wavelength of *C*_2_ is apparently lower than that of *C*_1_. The energy of light is highly concentrated in the high Q cavity (*C*_2_) at point *A*.

**Figure 13:**
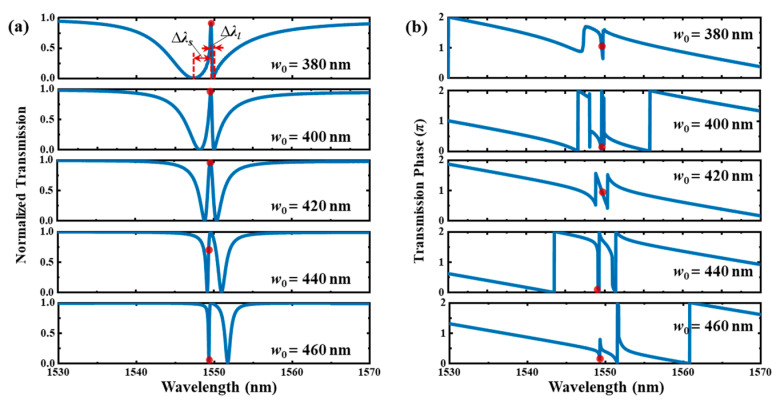
(**a**) The transmission spectra and (**b**) phase of the type Ⅱ dual-PCNC system with different bus waveguide widths calculated by the 3D FDTD method. The red dot is the point of the maximum slope change in phase relative to the wavelength.

Figure 13a shows the transmission spectra of the type Ⅱ system with different *w*_0_. When *w*_0_ decreases to 380 nm, the asymmetric EIT line shape occurs at 1549.6 nm. The degree of spectral asymmetry (*F*) is 8.54. Here, *F* is defined as ∆*λ_s_*/∆*λ_l_*, where ∆*λ_s_* and ∆*λ_l_* are the peak-to-dip bandwidths in the short and long wavelength directions, respectively [13]. The extinction ration and roll-off rate reach 22.7 dB and −156.3 dB/nm, respectively. When *w*_0_ increases to 460 nm, *F* decreases to 0.15. The curve near the dip of 1549.3 nm can be regarded as a Fano line shape. *q* is deduced to be about −5.89. The extinction ratio is 15.2 dB, and the roll-off rate reaches a high value of 258.1 dB/nm. It should be noted that the variation trend in the line shape caused by the effective index variation in the bus waveguide is opposite to the one described in Section 2 when the effective index of the bus waveguide becomes smaller.

The calculated transmission phases with different *w*_0_ are shown in Figure 13b. When the spectrum has a perfectly symmetric EIT-like line shape, the highest group delay point is located at the transmission peak, and the calculated group delay is 6.3 ps. When the line shape becomes asymmetric, this point will slightly deviate from the transmission peak.

**Figure 14:**
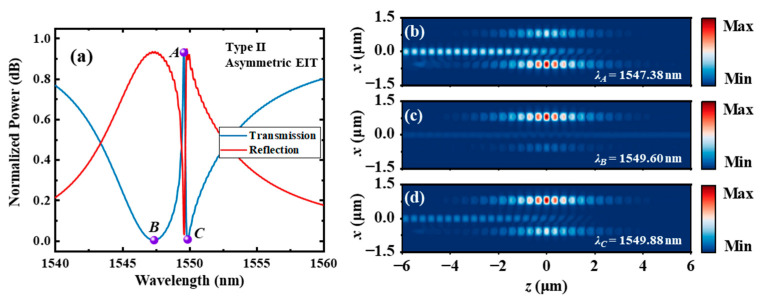
(**a**) The transmission and reflection spectra of the type Ⅱ dual-PCNC system with an asymmetric EIT line shape (*w*_0_ = 380) nm, and the |*H_y_*| profile at (**b**) point *A*, (**c**) point *B* and (**d**) point *C* calculated by the 3D FDTD method.

**Figure 15:**
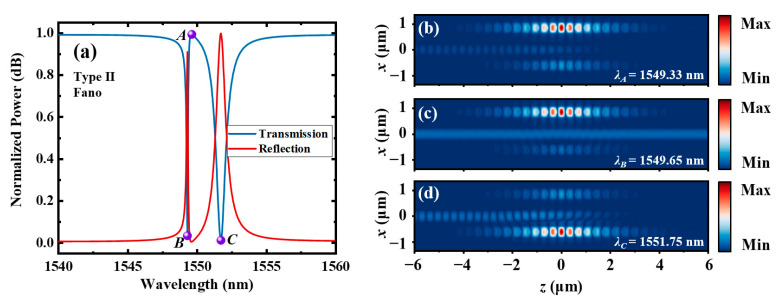
(**a**) The transmission and reflection spectra of the type Ⅱ dual-PCNC system with a Fano line shape and (*w*_0_ = 460), and the |*H_y_*| profile at (**b**) point *A*, (**c**) point *B* and (**d**) point *C* calculated by the 3D FDTD method.

The |*H_y_*| distributions of the type Ⅱ dual-PCNC system with an asymmetric EIT line shape (*w*_0_ = 380 nm) are also calculated. Figure 14b, c and d, respectively, show the |*H_y_*| distributions at points *A*, *B* and *C*, which are labeled in Figure 14a. This figure shows that the energy of light is mainly stored in *C*_1_ at point *A*, and it is mainly stored in *C*_2_ at points *B* and *C*. The |*H_y_*| distributions of magnetic fields of the type Ⅱ dual-PCNC system with a Fano line shape (*w*_0_ = 460) are shown in Figure 15. Figure 15b, c and d, respectively, show the |*H_y_*| distributions at points *A*, *B* and *C*, which are labeled in Figure 15a. This figure shows that the energy of light is mainly stored in *C*_1_ at point *C*, and it is mainly stored in *C*_2_ at points *A* and *B*.

## 4. Fabrication Error Analysis

In the following, we discuss the impact of fabrication errors including the deviation of waveguide width (∆*w*) and hole radius (∆*r*) on the transmission spectra of the type Ⅰ and type Ⅱ dual-PCNC systems with EIT-like line shapes.

**Figure 16:**
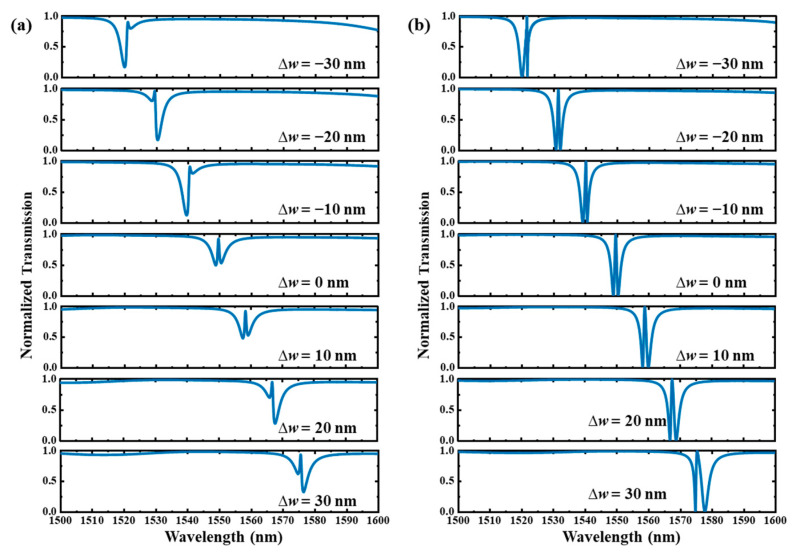
The transmission spectra of the (**a**) type Ⅰ and (**b**) type Ⅱ dual-PCNC systems with EIT line shapes with different waveguide width deviations (∆*w*).

Figure 16 shows the transmission spectra with waveguide width deviation (∆*w*) ranging from −30 μm to 30 μm. Here, the center positions of the waveguides remain unchanged, and the waveguide gaps are changed accordingly. It can be seen that the resonant peak is linearly shifted with a slope (∆*λ*/∆*w*) of about 0.92 nm/nm. For the type Ⅰ system, the line shape becomes slightly asymmetric with a positive *q* when ∆*w* > 0, as shown in Figure 16a. The line shape varies more dramatically when ∆*w* < 0. In addition, *q* is −0.85 when ∆*w* = −10 nm, and it then turns to a positive value of 0.65 when ∆*w* = −20 nm and subsequently becomes a negative value of −0.79 when ∆*w* = −30 nm. For the type Ⅱ system, the transmission peak becomes much wider when ∆*w* > 0, as shown in Figure 16b. A Fano line shape emerges near the dip at 1570.4 nm when ∆*w* = 30 nm. In addition, it becomes an asymmetric EIT line shape when ∆*w* = −10 nm, then turns to a symmetric EIT line shape when ∆*w* = −20 nm, and subsequently becomes an asymmetric EIT line shape with an *F* up to 9.35 when ∆*w* = −30 nm. We can see that the EIT line shape is more susceptible to disruption when ∆*w* < 0 for the type Ⅰ system, whereas for the type Ⅱ system, it becomes more susceptible when ∆*w* > 0.

**Figure 17:**
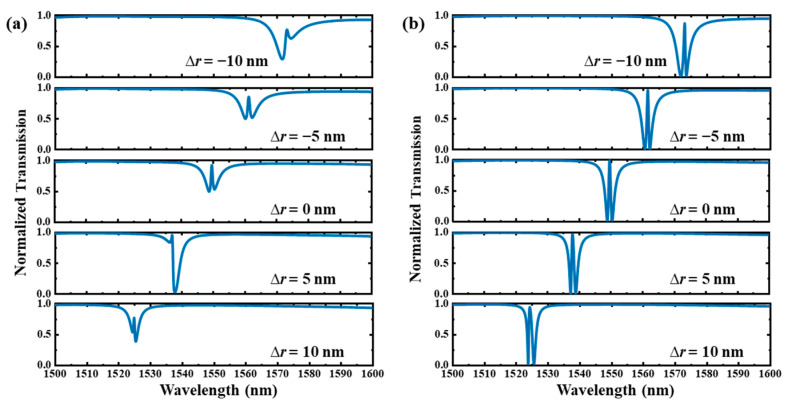
The transmission spectra of the (**a**) type Ⅰ and (**b**) type Ⅱ dual-PCNC systems with EIT line shapes with different hole radius deviations (∆*r*).

Figure 17 shows the transmission spectra with hole radius deviation (∆*r*) ranging from −10 μm to 10 μm. Here, we assume the radii of all the holes are changed with the same value. It can be seen that the resonant peak is linearly shifted with a slope (∆*λ*/∆*r*) of about −2.44 nm/nm. As shown in Figure 17a, for the type Ⅰ system, *q* reaches the highest value of 0.817 when ∆*r* = 5 nm. As shown in Figure 17b, for the type Ⅱ system, the transmission peak reaches an *F* of 2.17 when ∆*r* = −10 nm and reaches an *F* of 0.36 when ∆*r* = 10 nm. We can see that the EIT line shape of the type Ⅰ system is more susceptible to the influence of ∆*r* compared with the type Ⅱ system.

## 5. Conclusions

In conclusion, the dual indirectly side-coupled standing-wave cavities with 1D periodic structures are analyzed using CMT. Then, two types of dual-PCNC systems on the SOI platform are proposed and simulated using the 3D FDTD method. The EIT line shape can be achieved by carefully designing the bus waveguide width and the relative distance between *C*_1_ and *C*_2_. When the bus waveguide width is adjusted, the Fano line shape with a sharp edge occurs in the type Ⅰ system, and asymmetric EIT and Fano line shapes occur in the type Ⅱ system. The proposed structure with an EIT line shape has the advantages of low insertion loss, a high Q-factor and a wide FSR, which can be further used as optical sensor or filter. The proposed structures with Fano and asymmetric EIT line shapes can achieve high extinction ratios and high roll-off rates, qualifying them for further use as optical switches or modulators.

## Data Availability

The original contributions presented in this study are included in the article. Further inquiries can be directed to the corresponding author.

## References

[1] Li B.-B., Xiao Y.-F., Zou C.-L., Liu Y.-C., Jiang X.-F., Chen Y.-L., Li Y., Gong Q. (2011). Experimental observation of Fano resonance in a single whispering-gallery microresonator. Appl. Phys. Lett..

[2] Yang X., Yu M., Kwong D.-L., Wong C.W. (2009). All-Optical Analog to Electromagnetically Induced Transparency in Multiple Coupled Photonic Crystal Cavities. Phys. Rev. Lett..

[3] Zhang Z., Ng G.I., Hu T., Qiu H., Guo X., Wang W., Rouifed M.S., Liu C., Wang H. (2017). Conversion between EIT and Fano spectra in a microring-Bragg grating coupled-resonator system. Appl. Phys. Lett..

[4] Limonov M.F., Rybin M.V., Poddubny A.N., Kivshar Y.S. (2017). Fano resonances in photonics. Nat. Photonics.

[5] Miroshnichenko A.E., Flach S., Kivshar Y.S. (2010). Fano resonances in nanoscale structures. Rev. Mod. Phys..

[6] Miroshnichenko A.E., Kivshar Y.S. (2012). Fano Resonances in All-Dielectric Oligomers. Nano Lett..

[7] Hopkins B., Filonov D.S., Miroshnichenko A.E., Monticone F., Alù A., Kivshar Y.S. (2015). Interplay of Magnetic Responses in All-Dielectric Oligomers To Realize Magnetic Fano Resonances. ACS Photonics.

[8] Solodovchenko N.S., Samusev K.B., Limonov M.F. (2024). Fano resonances in all-dielectric nanostructures. All-Dielectric Nanophotonics.

[9] Yu Y., Heuck M., Hu H., Xue W., Peucheret C., Chen Y., Oxenløwe L.K., Yvind K., Mørk J. (2014). Fano resonance control in a photonic crystal structure and its application to ultrafast switching. Appl. Phys. Lett..

[10] Limonov M.F. (2021). Fano resonance for applications. Adv. Opt. Photonics.

[11] Cai L., Li S.-w., Xiang F.-c., Liu J., Liu Q. (2023). Fano resonance in whispering gallery mode microcavities and its sensing applications. Opt. Laser Technol..

[12] Zhang J., Leroux X., Durán-Valdeiglesias E., Alonso-Ramos C., Marris-Morini D., Vivien L., He S., Cassan E. (2018). Generating Fano Resonances in a Single-Waveguide Silicon Nanobeam Cavity for Efficient Electro-Optical Modulation. ACS Photonics.

[13] Piao X., Yu S., Park N. (2012). Control of Fano asymmetry in plasmon induced transparency and its application to plasmonic waveguide modulator. Opt. Express.

[14] Liu Q., Zeng D., Mei C., Li H., Huang Q., Zhang X. (2022). Integrated photonic devices enabled by silicon traveling wave-like Fabry-Perot resonators. Opt. Express.

[15] Zhang J., Cheng Z., Dong J., Zhang X. (2022). Cascaded nanobeam spectrometer with high resolution and scalability. Optica.

[16] Gu L., Wang B., Yuan Q., Fang L., Zhao Q., Gan X., Zhao J. (2021). Fano resonance from a one-dimensional topological photonic crystal. APL Photonics.

[17] Shi P., Zhou G., Deng J., Tian F., Chau F.S. (2015). Tuning all-Optical Analog to Electromagnetically Induced Transparency in nanobeam cavities using nanoelectromechanical system. Sci. Rep..

[18] Ciminelli C., Innone F., Brunetti G., Conteduca D., Dell’Olio F., Tatoli T., Armenise M.N. Rigorous model for the design of ultra-high Q-factor resonant cavities. Proceedings of the 2016 18th International Conference on Transparent Optical Networks (ICTON).

[19] Hardy A.A. (1998). A unified approach to coupled-mode phenomena. IEEE J. Quantum Electron..

[20] Barreda A., Mercadé L., Zapata-Herrera M., Aizpurua J., Martínez A. (2022). Hybrid Photonic-Plasmonic Cavity Design for Very Large Purcell Factors at Telecommunication Wavelengths. Phys. Rev. Appl..

[21] Chang C.-M., Solgaard O. (2013). Fano resonances in integrated silicon Bragg reflectors for sensing applications. Opt. Express.

[22] Cheng Z., Dong J., Zhang X. (2020). Ultracompact optical switch using a single semisymmetric Fano nanobeam cavity. Opt. Lett..

[23] Sun F., Li Z., Tang B., Li B., Zhang P., Liu R., Yang G., Huang K., Han Z., Luo J. (2023). Scalable high Q-factor Fano resonance from air-mode photonic crystal nanobeam cavity. Nanophotonics.

[24] Han Z., Wang C., Liu Y., Tian H. (2021). Simultaneous detection of complex refractive index and temperature using a compact side-coupled photonic crystal nanobeam cavity. J. Opt. Soc. Am. B.

[25] Yan Y., Jiang Y.-F., Li B.-X., Deng C.-S. (2024). Controlling Dual Fano Resonance Lineshapes Based on an Indirectly Coupled Double-Nanobeam-Cavity Photonic Molecule. J. Light. Technol..

[26] Meng Z.-M., Liang A., Li Z.-Y. (2017). Fano resonances in photonic crystal nanobeams side-coupled with nanobeam cavities. J. Appl. Phys..

[27] Yu P., Hu T., Qiu H., Ge F., Yu H., Jiang X., Yang J. (2013). Fano resonances in ultracompact waveguide Fabry-Perot resonator side-coupled lossy nanobeam cavities. Appl. Phys. Lett..

[28] Lin T., Chau F.S., Deng J., Zhou G. (2015). Dynamic control of the asymmetric Fano resonance in side-coupled Fabry–Pérot and photonic crystal nanobeam cavities. Appl. Phys. Lett..

[29] Dong G., Wang Y., Zhang X. (2018). High-contrast and low-power all-optical switch using Fano resonance based on a silicon nanobeam cavity. Opt. Lett..

[30] Jiang F., Deng C.-S., Lin Q., Wang L.-L. (2019). Simulation study on active control of electromagnetically induced transparency analogue in coupled photonic crystal nanobeam cavity-waveguide systems integrated with graphene. Opt. Express.

[31] Ma J., Deng C.-S., Lin Q., Wang L.-L. (2022). Graphene-based active tunable mode splitting in an indirectly coupled photonic crystal nanobeam cavity–waveguide system. J. Opt. Soc. Am. B.

[32] Manolatou C., Khan M., Fan S., Villeneuve P.R., Haus H., Joannopoulos J. (1999). Coupling of modes analysis of resonant channel add-drop filters. IEEE J. Quantum Electron..

[33] Li Q., Wang T., Su Y., Yan M., Qiu M. (2010). Coupled mode theory analysis of mode-splitting in coupled cavity system. Opt. Express.

[34] Yariv A. (1973). Coupled-mode theory for guided-wave optics. IEEE J. Quantum Electron..

[35] Zhao Y., Shi Y., Liu G., Dai P., Hao L., Ma Y., Liu S., Chen X. (2023). Study of resonant mode coupling in the transverse-mode-conversion based resonator with an anti-symmetric nanobeam Bragg reflector. Opt. Express.

[36] Zhao Y., Shi Y., Dai P., Liu S., Hao L., Ma Y., Chen X. (2022). Side-coupled Fabry-Perot resonator filter based on dual-waveguide Bragg grating. J. Light. Technol..

[37] Gu L., Fang L., Fang H., Li J., Zheng J., Zhao J., Zhao Q., Gan X. (2020). Fano resonance lineshapes in a waveguide-microring structure enabled by an air-hole. APL Photonics.

